# Gallic acid mitigates high-fat and high-carbohydrate diet-induced steatohepatitis by modulating the IRF6/PPARγ signaling pathway

**DOI:** 10.3389/fphar.2025.1563561

**Published:** 2025-04-01

**Authors:** Jiahao Qiu, Lihong Fu, Yan Xue, Yilan Yang, Fengjie Qiao, Wanchun Zhu, Yating Gao, Miao Fang, Yufei Liu, Zhujun Gao, Yunfeng Guan, Yueqiu Gao, Xin Zhang, Zhi Shang

**Affiliations:** ^1^ Institute of Infectious Disease, Shuguang Hospital Affiliated to Shanghai University of Traditional Chinese Medicine, Shanghai, China; ^2^ Laboratory of Cellular Immunity, Shuguang Hospital Affiliated to Shanghai University of Traditional Chinese Medicine, Shanghai, China; ^3^ Shanghai University of Traditional Chinese Medicine, Shanghai, China; ^4^ Department of Liver Diseases, Shuguang Hospital Affiliated to Shanghai University of Traditional Chinese Medicine, Shanghai, China

**Keywords:** gallic acid, steatohepatitis, pparγ signaling pathway, IRF6, lipid metabolism

## Abstract

Gallic acid (GA), a natural organic phenolic compound, is an abundant plant food bioactive substance present in many medicinal herbs. GA has anti-oxidative, anti-inflammatory and anticancer activities on multiple metabolic disorders. The present study was carried out to uncover the alleviating effects of GA on metabolic dysfunction-associated steatohepatitis (MASH) and the underlying mechanisms of its action. In this study, a mouse model of MASH induced by high-fat and high-carbohydrate diet was used to test the impact of GA on metabolic disorders. We found that GA administration attenuated obesity and fatty liver, relieved insulin resistance, and mitigated hepatic steatosis, inflammation and liver injury. Transcriptome sequencing (RNA-seq) of mouse liver tissues identified 154 differentially expressed genes (DEGs) among the NCD, HFHC, and GA groups. Bioinformatic analysis of these DEGs revealed significant enrichment in lipid metabolism function and the PPARγ signaling pathway, which were further validated. Overexpression of PPARγ significantly reduced the therapeutic effect of GA both *in vitro* and *in vivo*. Notably, the transcription factor interferon regulatory factor 6 (IRF6), a protective factor in metabolic stress, which was predicted as the upstream regulator, was significantly upregulated by GA. Furthermore, it was verified that GA’s anti-lipid deposition effect depends on the negative regulation of IRF6 on PPARγ using knocking-down strategy. Taken together, GA increases hepatic IRF6 expression, which mitigates lipid accumulation of hepatocytes and subsequent liver damage via inhibiting the PPARγ signaling pathway. These findings suggest a novel strategy for MASH management based on pharmacological intervention with GA.

## Graphical Abstract

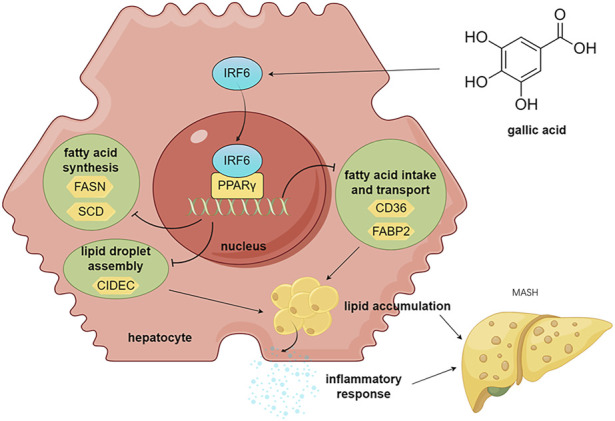

## 1 Introduction

Metabolic dysfunction-associated steatotic liver disease (MASLD) has become a global health concern, with its prevalence rising in parallel with increasing rates of obesity, type 2 diabetes, and metabolic syndrome. Approximately 25% of the world’s population is affected by MASLD (Friedman et al., 2018). Unfortunately, to date, there is a lack of therapeutic strategies for MASLD, which has spurred vigorous research efforts to identify pertinent pathophysiological mechanisms that can be targeted for new therapy development.

Dysfunction in lipid metabolism disrupts the intricate balance of processes involved in the synthesis, breakdown, and utilization of fat in the liver. This disruption results in excessive accumulation of lipids, particularly triglycerides, within hepatocytes, leading to hepatic steatosis, a predominant feature of MASLD (Badmus et al., 2022). Over time, if not controlled, fat deposition can lead to inflammation, liver injury, and ultimately more severe forms of MASLD, including metabolic dysfunction-associated steatohepatitis (MASH). Excessive hepatic lipid accumulation induces oxidative stress and consequent hepatocyte apoptosis, eventually leading to advanced liver fibrosis (Zambo et al., 2013; Canbay et al., 2004). Correcting lipid metabolism disorders has become a key focus in developing MASH therapies, which aim to restore the balance of lipid metabolism and limit liver damage (Xu et al., 2022).

Peroxisome proliferator-activated receptor gamma (PPARγ) is a nuclear receptor involved in adipogenesis, fat cell formation, and lipid accumulation (Grygiel-Gorniak, 2014). While its primary role is in adipose tissue, PPARγ is also expressed in other cell types, including hepatocytes (Tailleux et al., 2012). PPARγ plays a crucial role in regulating lipid metabolism as it affects many aspects of lipid metabolism. Its overexpression or excessive activation in hepatocytes results in an increased uptake of fatty acids from the bloodstream, enhanced synthesis of fatty acids within the liver, and reduced oxidation of fatty acids, all of which contribute to the accumulation of lipids in hepatocytes (Lee et al., 2018). The expression of PPARγ is increased in the livers of patients and mouse models with MASLD (Pettinelli and Videla, 2011; Nakamuta et al., 2005; Lee et al., 2021b). The loss of hepatocyte-specific expression of PPARγ (Pparg^ΔHep^) reduces diet-induced liver steatosis (Lee et al., 2021a; Wolf Greenstein et al., 2017) and slows the progression of steatohepatitis in mice fed a methionine/choline-deficient (MCD) diet (Cordoba-Chacon, 2020). Hence, targeting hepatic PPARγ presents a promising approach for MASH treatment.

Interferon regulatory factor 6 (IRF6), a member of the IRF family, plays pivotal roles in various biological processes, ranging from embryonic development to immune response regulation (Ingraham et al., 2006; Moretti et al., 2010). It functions as a transcription factor by binding to regulatory elements within the DNA of other genes to regulate their expression. In the immune response, IRF6 may regulate genes involved in cytokine production, inflammation, and antiviral responses (Huynh et al., 2016; Kwa et al., 2016; Li et al., 2016). During development, particularly orofacial development, IRF6 targets genes that control the formation and patterning of tissues, including the lip and palate (Dai et al., 2015). Studying IRF6’s target genes provides insight into their roles in multiple diseases, which is crucial for developing therapeutic strategies to correct or mitigate the effects of IRF6-related disorders.

Gallic acid (GA), chemically known as 3,4,5-trihydroxybenzoic acid, is a natural organic phenolic compound found in various herbal remedies (Oyagbemi et al., 2016). It possesses antioxidant, anti-inflammatory, and hepatoprotective properties, making it a subject of interest in research related to metabolic disorders, including obesity, type 2 diabetes, and hyperlipidemia (Kahkeshani et al., 2019). With the continuous expansion of research, GA is gradually being used as an experimental treatment for MASLD. Recent studies have suggested that GA mitigates hepatic steatosis by activating the AMP-activated protein kinase (AMPK) signaling cascade (Tanaka et al., 2020; Zhang et al., 2023; Lu et al., 2023). Moreover, GA ameliorated hepatic steatosis and inflammation in an MCD diet-induced MASH animal model (Tung et al., 2018). However, in the context of MASH, the potential molecular mechanism underlying the pharmacological activity of GA remains to be explored through more intensive studies.

Consequently, this study aimed to perform RNA sequencing (RNA-seq) analysis of the livers of a high-fat, high-carbohydrate (HFHC) diet-induced MASH mouse model and conduct validation experiments to identify the potential targets and pathways of GA action. These results provide new insights into the mechanisms underlying the pharmacological effects of GA on MASH and may contribute to novel therapeutic strategies for the management of MASH.

## 2 Materials and methods

### 2.1 Animals and treatments

Seven-week-old male C57BL/6J mice, purchased from Shanghai Jihui Experimental Animal Breeding Co., Ltd. (Shanghai, China), were fed adaptively for 1 week. To establish a MASH mouse model, the mice were fed an HFHC diet with a high-fat diet (60% kcal from fat, D12492i, Research Diets, USA) and a high fructose-glucose solution (23.1 g/L d-fructose and 18.9 g/L d-glucose, F0001, S0001, Trophic Animal Feed High-tech Co., Ltd., China). After 16 weeks, GA was administered daily via oral gavage at doses of 50 mg/kg (low-dose) and 200 mg/kg (high-dose). Metformin (100 mg/kg) was administered daily via oral gavage. HFHC mice were administered normal saline daily via oral gavage. All animal experimental protocols were approved by the Institutional Animal Care and Use Committee of Shanghai Traditional Chinese Medicine University (PZSHUTCM2302080004 approved on 06/12/2022).

### 2.2 Insulin tolerance test (ITT) and glucose tolerance test (GTT)

For the ITT, mice were injected intraperitoneally with insulin solution (1 mU/g body weight) after a 6 h fast. For the GTT, mice were injected intraperitoneally with glucose solution (1 mg/g body weight) after a 16-h fast. Blood glucose levels were recorded at 0, 30, 60, 90, and 120 min in blood samples obtained from the tail tip.

### 2.3 Hematoxylin-eosin (HE) and oil red O staining

To investigate histological change, liver tissue was embedded in paraffin, cut into 5 μm serial sections, and then subjected to standard HE staining. For the determination of hepatic fat accumulation, 8 μm frozen liver sections were stained with Oil Red O according to standard methods. To analyze intracellular lipid accumulation, HepG2 cells in 6-well plates were washed with phosphate-buffered saline (PBS), fixed with 10% neutral formalin, and then stained with Oil Red O and hematoxylin.

### 2.4 Immunohistochemistry assay

For immunohistochemical staining, liver sections were incubated with the primary antibody against F4/80 (1:8,000, 29414-1-AP, Proteintech) at 4°C overnight, followed by incubation with appropriate horseradish peroxidase (HRP)-labeled secondary antibody. After the 3,3′-diaminobenzidine (DAB) chromogenic reaction, the slides were washed and mounted for microscopic examination.

### 2.5 Serum and liver measurements

Serum triglyceride, alanine aminotransferase (ALT), and aspartate aminotransferase (AST) were determined using an automatic biochemical analyzer. Serum interleukin-6 (IL-6), interleukin-1β (IL-1β), tumor necrosis factor-α (TNF-α), interferon-γ (IFN-γ), and interleukin-17A (IL-17A) levels were detected using corresponding enzyme-linked immunosorbent assay kits according to the manufacturer’s protocol (MultiSciences, China). Triglyceride levels in the liver and hepatocyte samples were measured using commercial kits (E1013, Applygen, Beijing, China).

### 2.6 Cell culture

HepG2 hepatocytes were cultured in DMEM (high glucose, with pyruvate, L-glutamine) (Meilunbio, Dalian, China) supplemented with 10% fetal bovine serum (Thermo Scientific, Waltham, MA, USA) and 1% penicillin-streptomycin. The cells were cultured at 37°C in a humidified incubator with 5% CO_2_.

### 2.7 Cell counting kit-8 (CCK-8) assay

HepG2 cells were cultured in 96-well plates at a density of 5 × 10^3^ cells/well overnight, followed by treatment with different concentrations of GA (0–180 μM) for 24 h. Subsequently, 10 μl CCK-8 solution was added to each well. After culturing in a cell incubator at 37°C for 2 h, a microplate reader (Biotek, Vermont, United States) was used to measure the absorbance at 450 nm for each sample well.

### 2.8 RNA sequencing and bioinformatic analysis

Liver tissue samples were collected from mice in the NCD group, HFHC group, and GAH group, with 3 biological replicates set for each group. Total liver RNA was extracted, and cDNA libraries were constructed to profile the differences in gene expression. The NovaSeq™ 6000 platform (Illumina) was used for paired-end sequencing of the libraries. HISAT2 software (version 2.2.1) was used to map the reads to Ensembl mice (mm10/GRCm38). SAM tool (version 0.1.19) was employed to generate a binary alignment map. StringTie (version 2.1.6) was used to calculate the raw gene counts. To normalize the count matrix, the DESeq2 software was applied. The criteria for identifying differentially expressed genes (DEGs) were as follows: genes with a fold change (FC) ≥ 2 or ≤0.5 (log_2_FC ≥ 1 or ≤ −1) and a false discovery rate (FDR)-adjusted *p*-value <0.05 were considered significantly differentially expressed.

### 2.9 Quantitative reverse-transcription-polymerase chain reaction (qRT-PCR)

Total RNA was extracted separately from the liver tissue and cells using an RNA quick purification kit (SB-R001, ShareBio, Shanghai, China) and then reverse transcribed into cDNA using a reverse transcription kit (R223-01, Vazyme, Nanjing, China) according to the manufacturer’s instructions. SYBR qPCR master mix (Q711-02; Vazyme, Nanjing, China) was used to perform real-time quantitative PCR (qPCR) according to the manufacturer’s protocol. The relative mRNA expression levels of the target genes were normalized to those of the housekeeping gene glyceraldehyde-3-phosphate dehydrogenase. The primer pairs used are listed in Supplementary Table 1.

### 2.10 Western blot

Total protein was extracted using a radio-immunoprecipitation assay (RIPA) lysis solution (SB-BR040; ShareBio, Shanghai, China) containing protease and phosphatase inhibitors. Total protein was quantified using a bicinchoninic acid assay kit (SB-WB013; ShareBio, Shanghai, China). Protein samples were mixed with 5× loading buffer, subjected to sodium dodecyl sulfate-polyacrylamide gel electrophoresis, and transferred to a polyvinylidene fluoride membrane. The proteins on the membrane were incubated with the appropriate primary antibody overnight at 4°C, followed by the corresponding secondary antibody for 1 h at room temperature. Signals were visualized using enhanced chemiluminescence (SB-WB011; ShareBio, Shanghai, China). The primary antibodies used are listed in Supplementary Table 2.

### 2.11 Immunofluorescence assay

For immunofluorescence staining, the cells were fixed with 4% paraformaldehyde for 15 min. Subsequently, the cells were permeabilized with 0.1% Triton X-100 at room temperature for 10 min. After being blocked with 10% goat serum for 1 h at 37°C, the cells were incubated with the primary antibody of PPARγ (1:200, 66936-1-Ig, Proteintech) overnight at 4°C. Subsequently, the cells were incubated with the corresponding secondary antibody for 1 h at room temperature, and the signals were examined under a fluorescence microscope (IX71, Olympus, Japan).

### 2.12 Lentivirus (LV) infection

To overexpress the target gene, HepG2 cells were seeded in 6-well plates at > 60% confluence and used for lentiviral infection. LV containing pLV11ltr-Puro-mCherry-CMV-PPARG was constructed and purchased from Beijing Tsingke Biotech Co., Ltd. According to the manufacturer’s instructions, LV was added at a multiplicity of infection of 10, and the medium was replaced after 8 h. After puromycin was added for 3 days, the cells were used for further experiments.

### 2.13 Small-interfering RNA (siRNA) transfection

To knockdown the target gene, HepG2 cells were transfected with two siRNAs (GenePharma Shanghai, China) specifically targeting IRF6 using Lipofectamine RNAiMAX (13,778-075, Invitrogen), according to the manufacturer’s instructions. The sequences of siRNAs against IRF6 were as follows: siIRF6-1(5′-3′): AUC​GCU​AAG​GAA​UGU​UUC​CTT. siIRF6-2(5′-3′): GGA​AAC​AUU​CCU​UAG​CGA​UTT.

### 2.14 Adeno-associated virus (AAV) transfection

Adenovirus carrying pAAV-TBG-Pparg-P2A-EGFP plasmid vector was purchased from Shanghai Bio-lifespan Co., LTD. for *in vivo* overexpression. Mice were intravenously injected with the adenoviral constructs (1 × 10^10^ IFU/mL) in the caudal vein once. After the injection, mice were monitored for any adverse reactions and allowed to recover under controlled conditions.

### 2.15 Statistical analysis

Data are expressed as mean ± standard error of the mean. Student’s *t*-tests were used to analyze the differences between the two groups. The multiple group comparisons were conducted using one-way analysis of variance (ANOVA), and pairwise comparisons between groups were performed using the LSD-*t* test. A *p*-value less than 0.05 was considered statistically significant. GraphPad Prism (version 9.5.0) was used for statistical analyses.

## 3 Results

### 3.1 GA attenuates fatty liver and improves insulin resistance (IR) induced by HFHC-diet in mice

**FIGURE 1:**
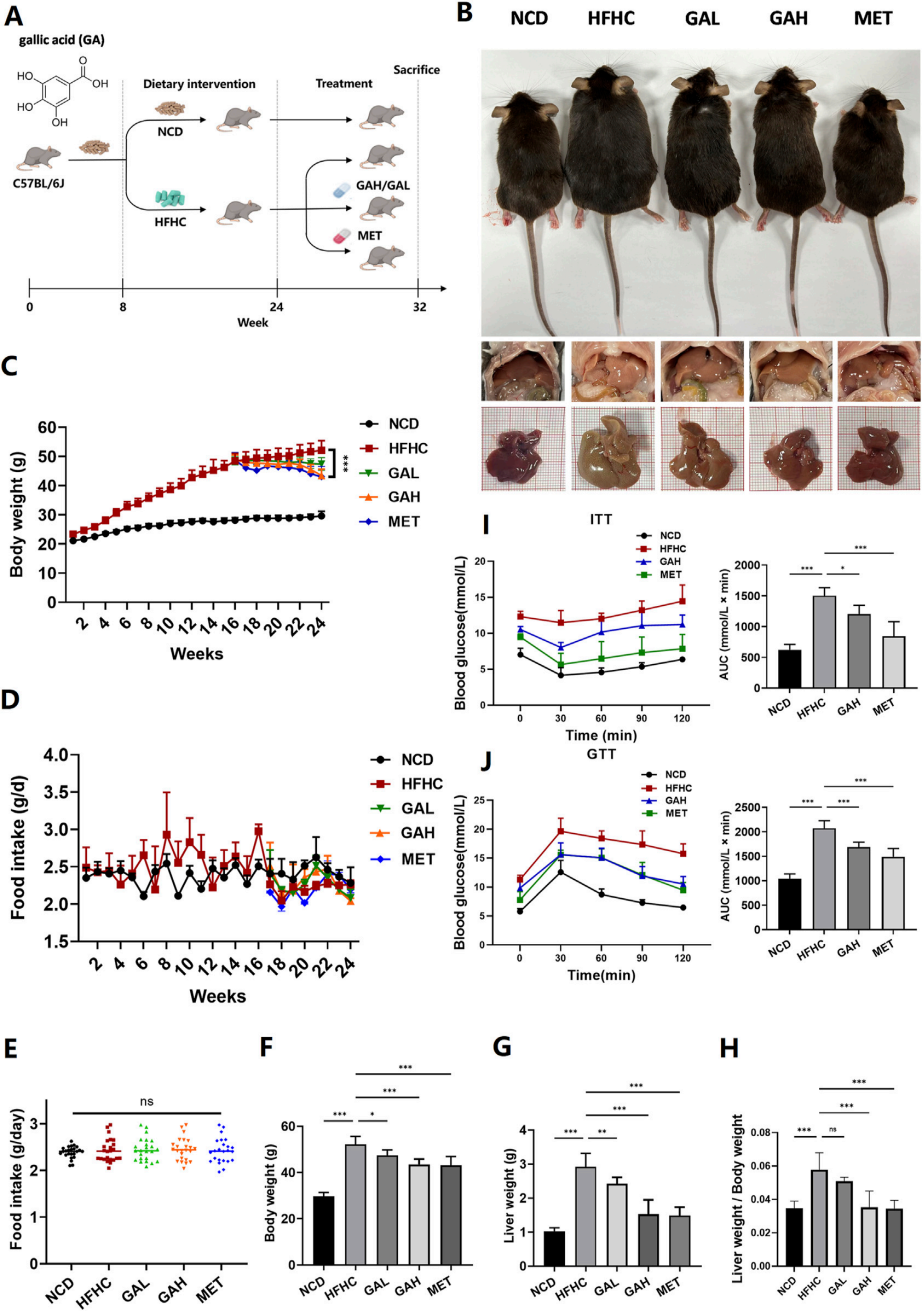
GA attenuates high-fat high-carbohydrate diet-induced fatty liver and improves insulin resistance in mice. **(A)** Experimental strategy of HFHC-induced MASH mouse model. HFHC-fed mice were given PBS, GA (low dose: 50 mg/kg, high dose: 200 mg/kg) or MET for 8 weeks starting at week 16. **(B)** Representative photos of liver and body size, and liver gross morphology of mice in each group. **(C)** Body weight change of mice in each group (n = 8 mice per group). **(D–H)** Food intake change, final body weight, liver weight, liver weight/body weight of mice in each group (n = 8 mice per group). **(I)** Insulin tolerance and **(J)** glucose tolerance test of mice in each group (n = 8 mice per group). The data are presented as the mean ± SEM. *p < 0.05, **p < 0.01, ***p < 0.001.

We established an HFHC diet-induced C57BL/6 mouse MASH model to examine the effects of GA on MASH. Mice were fed an HFHC diet for 16 weeks to induce steatohepatitis and obesity and were intragastrically administered GA at a high dose of 200 mg/kg (GAH) or a low dose of 50 mg/kg (GAL) daily while continuing to receive the HFHC diet for an additional 8 weeks. The doses of GA used in this study were based on previous studies showing its efficacy and safety in murine MAFLD models, where GA improved hepatic steatosis, inflammation, and fibrosis without adverse effects (Zhang et al., 2023). Mice used as positive controls were gavaged with metformin (MET), which has been widely reported to ameliorate hepatosteatosis and liver dysfunction in mice (Fullerton et al., 2013; Lin et al., 2017). Normal chow diet (NCD)-fed mice served as controls (Figure 1A). Body weight increased significantly in the HFHC-fed mice at 16 weeks (Figure 1C). At the end of the feeding period, compared with NCD-fed mice, HFHC-fed mice developed obesity and fatty liver disease. These changes were markedly reduced in the GA and MET groups (Figure 1B). After GA and MET treatments, the mice showed significant weight loss, and comparable effects on weight loss were observed between the GAH and MET groups (Figure 1C, F). GA intervention did not affect mice diets (Figure 1D–E), indicating that weight loss did not result from food intake. Additionally, GAH mice showed significantly decreased liver weight and liver/body ratio compared to the HFHC group, whereas GAL mice only showed a decreasing trend in the liver/body ratio (Figure 1G, H), indicating that GA reduces fatty liver in a dose-dependent manner.

Given that IR and its implications are strongly associated with the development of MASLD (Wu et al., 2021), we performed an ITT and a GTT in mice to investigate the effect of GA on IR. By calculating the area under the curve in the GTT and ITT assays, it was obvious that high-dose GA significantly prevented glucose and insulin tolerance in HFHC mice, although it was slightly inferior to MET (Figure 1I, J). Collectively, these findings demonstrate that GA administration can attenuate HFHC-induced fatty liver disease and improve IR.

### 3.2 GA administration alleviates hepatic steatosis, inflammation, and liver injury

**FIGURE 2:**
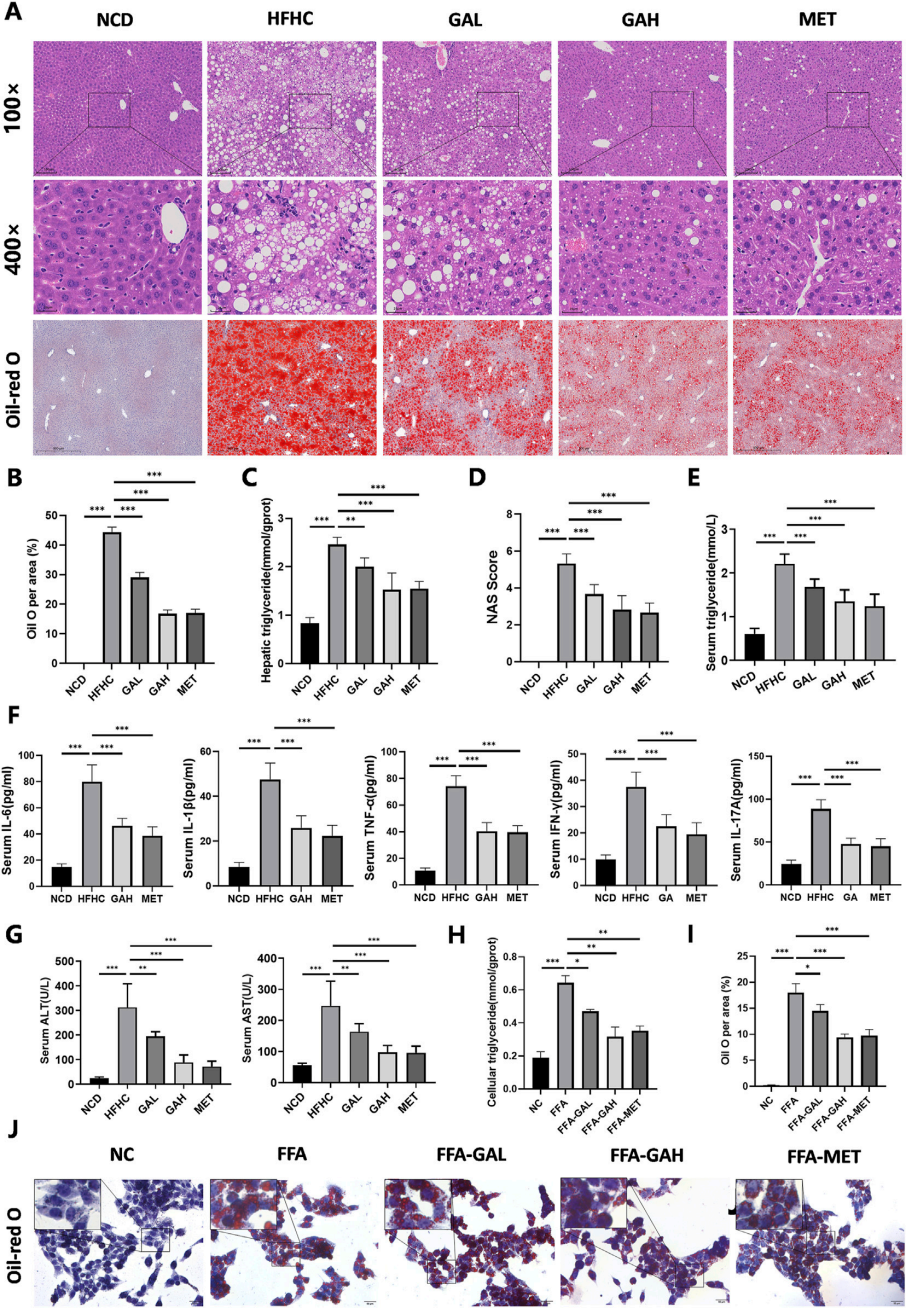
GA administration alleviates HFHC-induced hepatic steatosis, inflammation and liver injury. **(A)** Representative images of HE and Oil Red O staining of liver sections. **(B)** Quantification of Oil red O positive area per field (n = 5). **(C)** Liver triglyceride content of mice in each group (n = 5). **(D)** NAS score of liver sections in each group (n = 5). **(E–G)** The levels of triglyceride, IL-6, IL-1β, TNF-α, IFN-γ, IL-17A, ALT and AST in the serum of mice in each group (n = 8). **(H)** Cellular triglyceride content of HepG2 cells treated with FFA, GAL, GAH, or MET (n = 3). **(I)** Quantification of Oil red O positive area per field (n = 3). **(J)** Oil red O staining of HepG2 cells treated with FFA, GAL, GAH, or MET (n = 3). Scale bar: 100 μm. The data are presented as the mean ± SEM. *p < 0.05, **p < 0.01, ***p < 0.001.

Histologically, liver HE staining showed that HFHC diet-fed mice displayed pronounced steatosis and characteristic inflammatory cell infiltration, which were significantly improved in GA-treated mice (Figure 2A). Additionally, Oil Red O staining and triglyceride detection together indicated excessive lipid accumulation in the liver tissues of HFHC diet-fed mice, and high-dose GA therapy significantly reversed this exacerbating effect (Figure 2A–2C). Immunohistochemical staining of F4/80 revealed that GA treatment significantly reduced the number of macrophages in the livers (Supplementary Figure 1). Furthermore, the NAFLD activity score (NAS) was significantly higher in the HFHC-fed group but decreased in a dose-dependent manner in the GA-treated groups (Figure 2D). Similar to hepatic triglyceride levels, serum triglyceride levels in the GAH group were much lower than those in the HFHC group (Figure 2E). These therapeutic effects were also observed in the MET group, although there was no significant difference between the GAH and MET groups.

We investigated the effects of GA treatment on inflammatory damage in MASH mice. Quantitative detection of mouse serum pro-inflammatory factors (IL-6, IL-1β, TNF-α, IFN-γ, and IL-17A) exhibited notable repression in the GAH and the MET mice (Figure 2F). Similarly, the degree of HFHC diet-induced liver injury, as measured by serum ALT and AST levels, significantly decreased in the GAH and MET groups (Figure 2G). Collectively, these results suggest that GA protects the liver from inflammation and injury.

Given that hepatocytes constitute the primary cellular framework of the liver and are chiefly responsible for lipotoxicity induced by fatty acids, we were interested in whether GA acts directly on hepatocytes to exert its effects. We first conducted a CCK-8 assay to test the toxicity to human HepG2 cells when treated with different concentrations of GA for 24 h and observed that GA displayed an apparent inhibitory effect at a concentration of more than 60 μM on HepG2 cells (Supplementary Figure 1). Consequently, 20 μM (low-dose) and 40 μM (high-dose) were selected as the concentrations of GA for subsequent experiments. HepG2 cells were then challenged with free fatty acids (FFA, palmitic acid/oleic acid, 1 mM) for 24 h to establish an *in vitro* steatosis model and subsequently treated with GA at low (FFA-GAL) and high (FFA-GAH) doses and metformin (FFA-MET, the positive control) for another 24 h. To further validate the anti-lipid deposition effects of GA on hepatocytes under metabolic stress, we stained lipid droplets with Oil Red O and quantified triglyceride levels using spectrophotometry in HepG2 cells. Compared to bovine serum albumin (BSA)-treated control cells, FFA-treated cells showed much more severe lipid accumulation, which was decreased by GA treatment in a dose-dependent manner (Figure 2H–J). Notably, the effects on lipid accumulation were similar in the high-dose GA and MET groups. Altogether, these results demonstrate that GA inhibits hepatic lipid accumulation.

### 3.3 RNA-seq analysis of GA in the treatment of MASH

**FIGURE 3:**
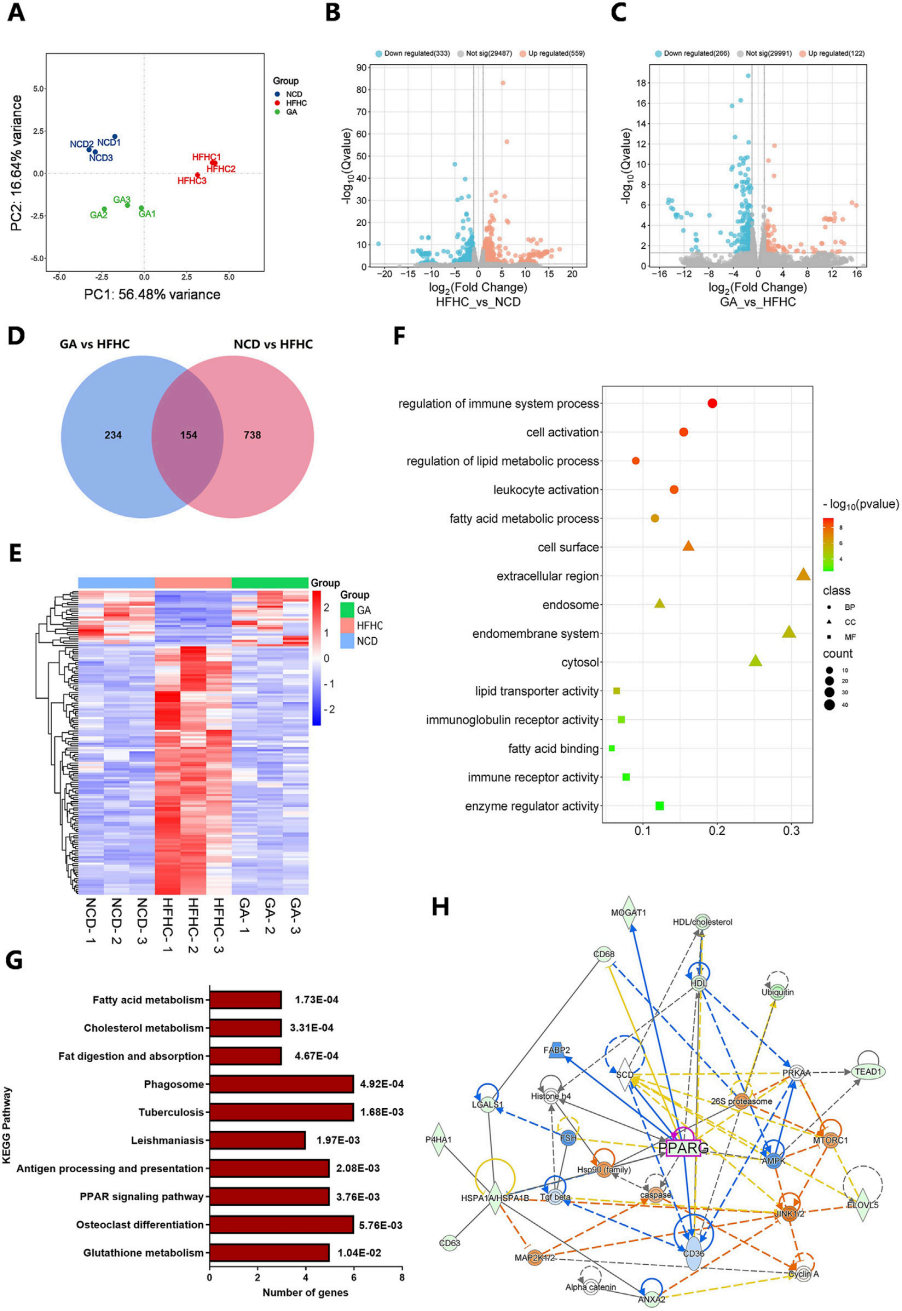
Transcriptome sequencing of livers from HFHC-fed and GA-treated mice and bioinformatic analysis of DEGs. **(A)** Principal component analysis (PCA) for transcriptome of all liver samples. **(B)** The volcano of DEGs between NCD and HFHC groups. **(C)** The volcano of DEGs between HFHC and GA groups. **(D)** Venn diagrams representing the overlap of the DEGs in NCD vs. HFHC and HFHC vs. GA. **(E)** The heatmap of DEGs in NCD, HFHC and GA groups. **(F)** GO enrichment analysis of DEGs. **(G)** KEGG pathway analysis of DEGs. **(H)** Protein-protein interaction network of DEPs constructed by IPA.

To investigate the mechanism of action of GA in MASH, we performed RNA-Seq analyses of the livers of NCD, HFHC, and GAH mice. Principal component analysis of gene expression profiles across all samples revealed distinct global transcriptome patterns among the three groups (Figure 3A). After quantification, 892 DEGs were identified between the NCD and HFHC group mice, with a fold change >2 and p-value <0.05 (Figure 3B). Overall, 388 DEGs were identified between the GAH and HFHC groups (Figure 3C). Additionally, 154 DEGs overlapped between the three groups (Figure 3D). Among them, 129 genes were downregulated and 25 genes were upregulated in GA-treated mice compared to those in the HFHC group (Supplementary Table 3). Figure 3E shows a heat map of the DEGs.

Furthermore, we performed Gene Ontology (GO) analysis of the DEGs. Biological process enrichment analysis revealed that the DEGs were involved in fatty acid metabolic processes and immune system regulation, which are key functions involved in lipid metabolism and inflammation related to MASH (Figure 3F). In the cellular components category, the extracellular region and endomembrane system were significantly enriched. Within the molecular function category, enzyme regulation and immune receptor activities were enriched. These results indicate that the main role of GA in MASH treatment is to regulate hepatic lipid metabolism and inflammation.

Kyoto Encyclopedia of Genes and Genomes (KEGG) pathway enrichment analysis revealed that metabolic pathways were mainly enriched, including fatty acid metabolism, cholesterol metabolism, glutathione metabolism, and the PPAR signaling pathway (Figure 3G). The protein-protein interaction network constructed using ingenuity pathway analysis (IPA) revealed interactions between the differentially expressed proteins (DEPs) involved in the regulation of lipid metabolism, including PPARG, AMPK, CD36, SCD, and FABP2 (Figure 3H).

### 3.4 GA downregulates the PPARγ signaling pathway

**FIGURE 4:**
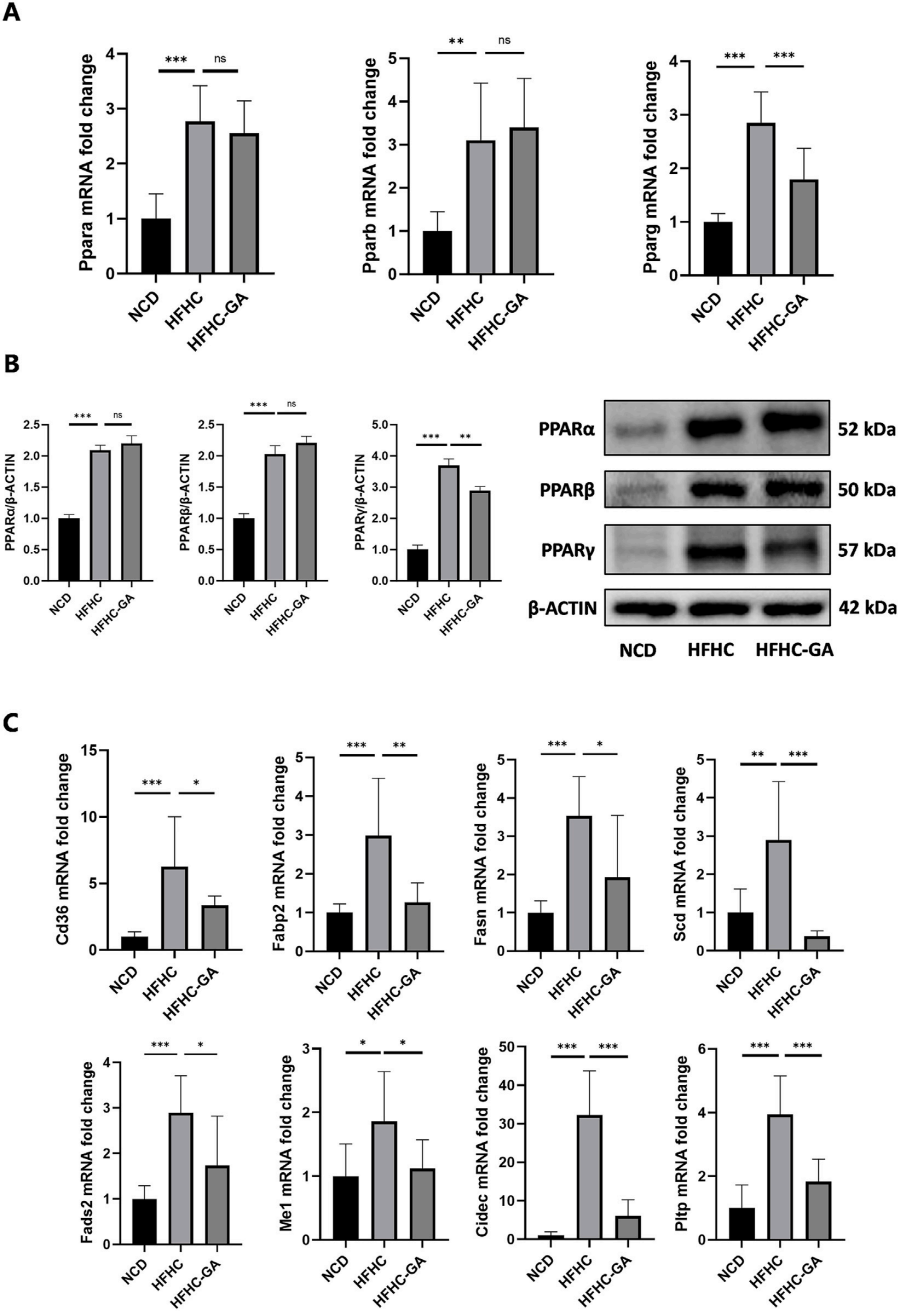
GA decreased the signaling transduction of PPARγ, instead of PPARα or PPARβ. **(A, B)** Analysis of expression of three subtypes of PPARs (PPARα, PPARβ, and PPARγ) in the livers of NCD, HFHC, and GA mice by qPCR (n = 3 per group) and Western blot (n = 3 per group). **(C)** The mRNA expression levels of CD36, FABP2, FASN, SCD, FADS2, ME1, CIDEC and PLTP in the livers of mice in each group (n = 3 per group). The data are presented as the mean ± SEM. *p < 0.05, **p < 0.01, ***p < 0.001.

**FIGURE 5:**
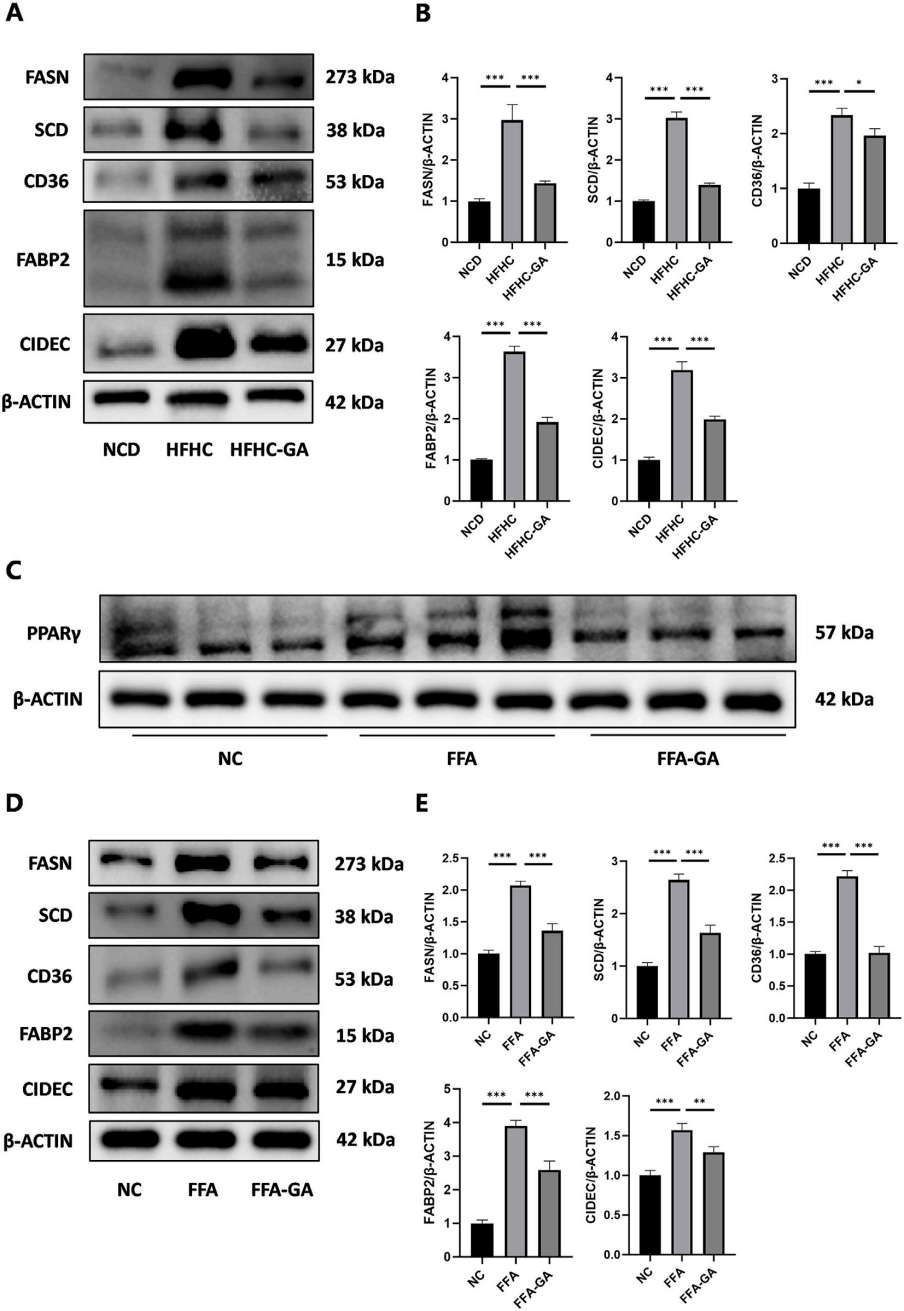
GA downregulated the levels of key proteins in the PPARγ pathway. **(A, B)** Analysis of FASN, SCD, CD36, FABP2, and CIDEC expression by Western blot in the livers of NCD, HFHC, GA mice (n = 3 per group). **(C)** Analysis of PPARγ expression by Western blot in HepG2 cells after FFA and FFA-GA treatment for 24 h, respectively (n = 3 per group). **(D, E)** Analysis of FASN, SCD, CD36, FABP2, and CIDEC expression by Western blot in FFA-induced HepG2 cells treated with GA (n = 3 per group). The data are presented as the mean ± SEM. *p < 0.05, **p < 0.01, ***p < 0.001.

Because the dysregulated PPAR signaling pathway may significantly contribute to excessive lipid accumulation within hepatocytes, leading to hepatic steatosis and inflammation, we focused on studying how GA modulates hepatic lipid metabolism through the PPAR signaling pathway. q-PCR and Western blot analysis were conducted to examine the expression of the three subtypes of PPARs (PPARα, PPARβ, and PPARγ) in mice and further confirmed GA’s influence on the PPAR signaling pathway. GA significantly reduced the PPARγ mRNA and protein expression, whereas the mRNA and protein levels of PPARα and PPARβ remained unaltered (Figure 4A, B). Given the pro-adipogenic effect mediated by PPARγ overexpression, we assumed that GA downregulates PPARγ signaling, thereby regulating lipid metabolism and reducing lipid droplet accumulation, which was verified via detecting several downstream genes, regulated by PPARγ, responsible for different stages of lipid metabolism. GA treatment significantly decreased the transcript levels of CD36 (involved in fatty acid intake), FABP2 (involved in fatty acid transport), FASN, SCD, FADS2, ME1 (involved in fatty acid biosynthesis), CIDEC (involved in lipid droplet assembly), and PLTP (involved in cholesterol transport) in the liver tissue (Figure 4C). Additionally, a significant reduction in the translation levels of CD36, FABP2, FASN, SCD, and CIDEC was observed after GA treatment (Figure 5A, B). Consistent with the *in vivo* findings, Western blot analysis of PPARγ and its target proteins CD36, FABP2, FASN, SCD, and CIDEC showed a notable decrease in their protein production in GA-treated HepG2 cells (Figure 5C–5E). These findings suggest that GA administration downregulates the PPARγ signaling pathway.

### 3.5 GA attenuates hepatocyte steatosis via a PPARγ-dependent mechanism

**FIGURE 6:**
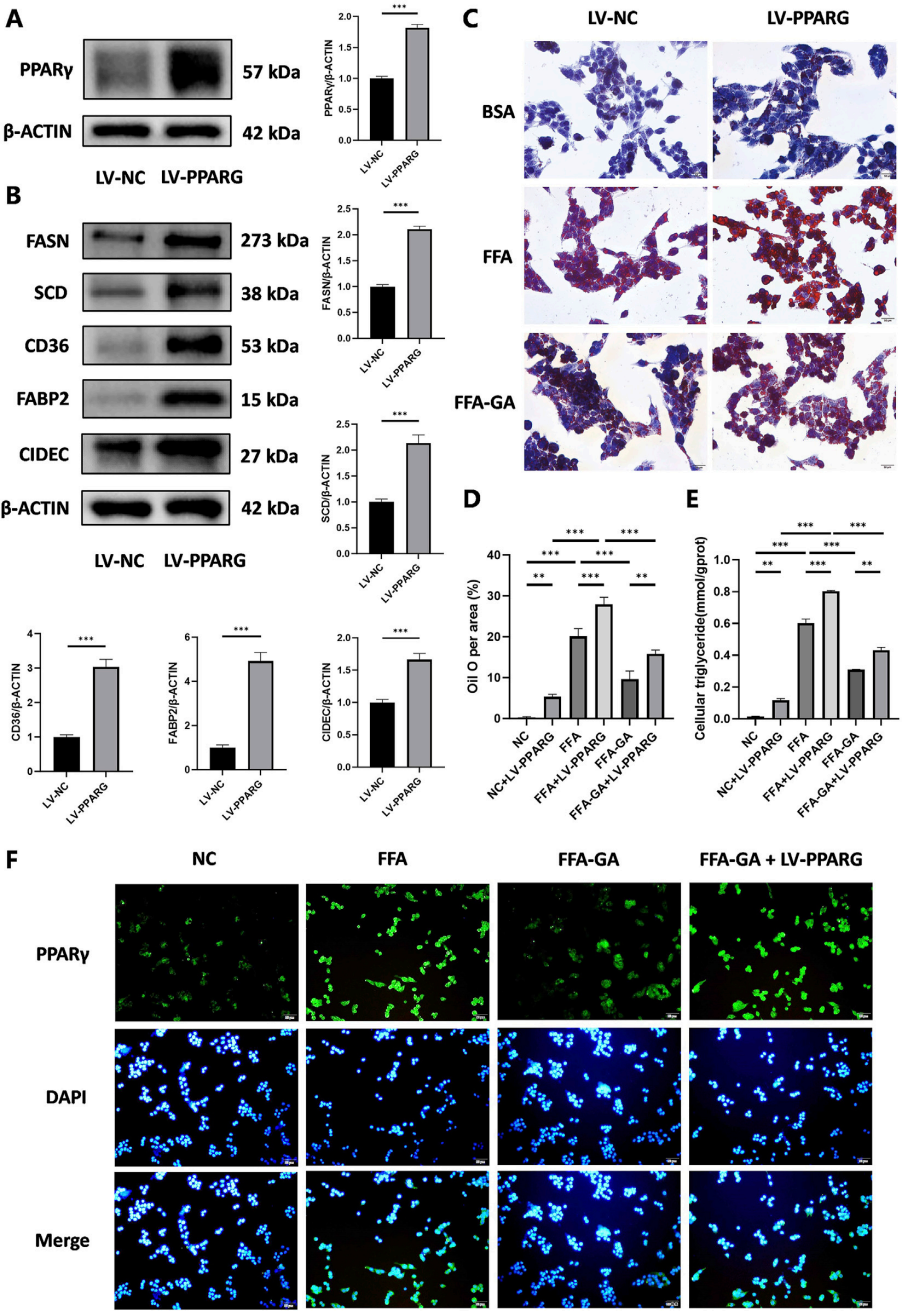
Inhibition of lipid accumulation effect of GA depends on the PPARγ signaling pathway. **(A)** Analysis of PPARγ expression by Western blot in PPARγ-overexpressing HepG2 cells (n = 3 per group). **(B)** Analysis of FASN, SCD, CD36, FABP2, and CIDEC expression by Western blot in PPARγ-overexpressing HepG2 cells (n = 3 per group). **(C)** Oil red O staining of PPARγ-overexpressing HepG2 cells treated with FFA and FFA-GA, respectively (n = 3 per group). **(D)** Quantification of Oil red O positive area per field (n = 3 per group). **(E)** Triglyceride levels in PPARγ-overexpressing HepG2 cells treated with FFA and FFA-GA, respectively (n = 3 per group). **(F)** Immunofluorescent staining of PPARγ of PPARγ-overexpressing HepG2 cells treated with FFA and FFA-GA, respectively (n = 3 per group). Scale bar: 100 µm. The data are presented as the mean ± SEM. *p < 0.05, **p < 0.01, ***p < 0.001.

**FIGURE 7:**
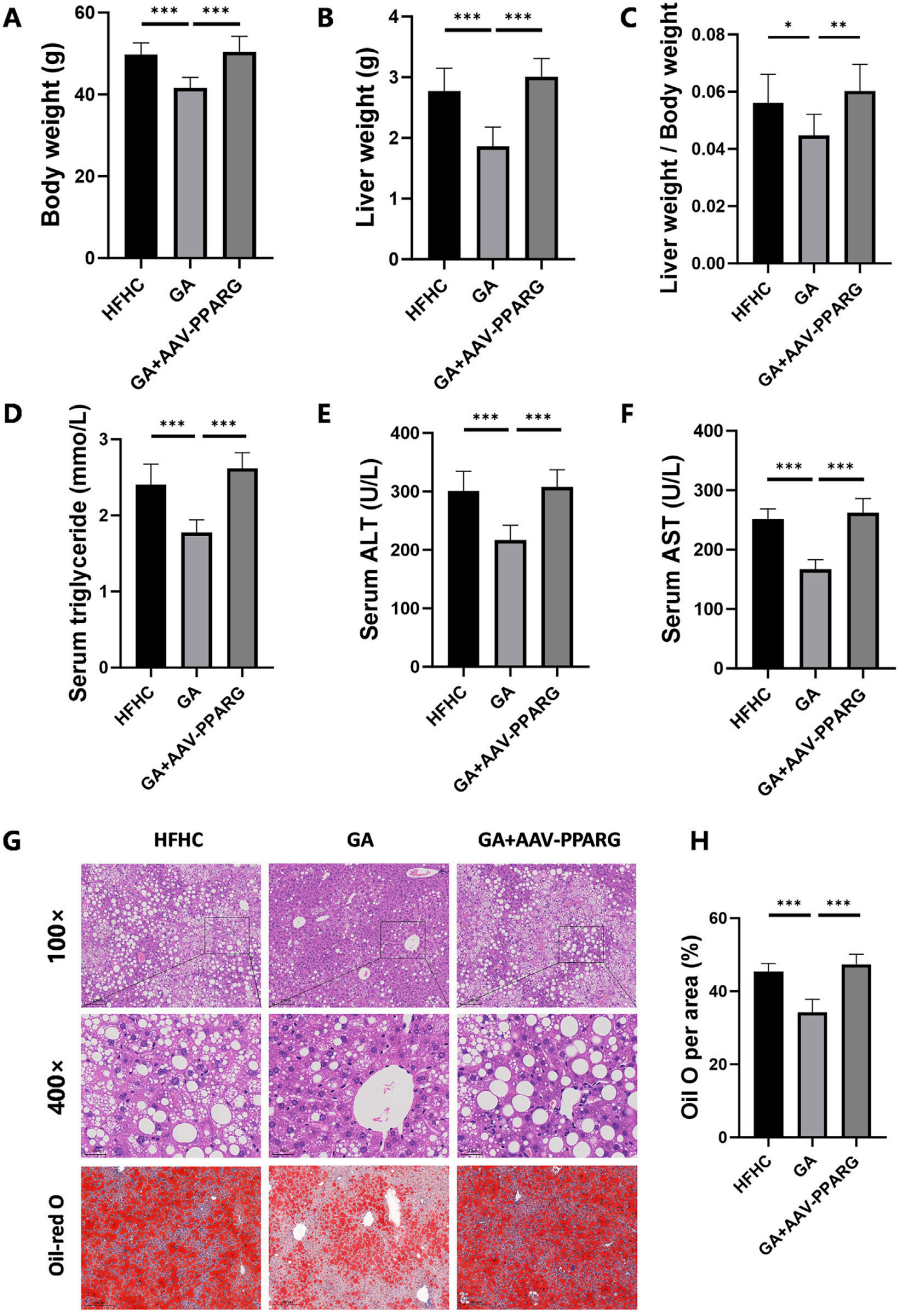
PPARγ overexpression counteracts the therapeutic effects of GA in MASH. **(A)** Final body weight, **(B)** liver weight, **(C)** liver weight/body weight of mice in the HFHC, GA, and GA+AAV-PPARG group (n = 8 mice per group). **(D–F)** The levels of triglyceride, ALT, and AST in the serum of mice in each group (n = 8). **(G)** Representative images of HE and Oil Red O staining of liver sections. **(H)** Quantification of Oil red O positive area per field (n = 3). Scale bar: 100 μm. The data are presented as the mean ± SEM. *p < 0.05, **p < 0.01, ***p < 0.001.

To identify whether the anti-lipid accumulation effects of GA in hepatocytes depend on the PPARγ signaling, HepG2 cells were infected with an LV delivering pLV11ltr-Puro-mCherry-CMV-PPARG (LV-PPARG) to obtain a stably PPARγ-overexpression cell line. The efficiency of LV-PPARG overexpression was validated using Western blotting (Figure 6A). As expected, the protein levels of CD36, FABP2, FASN, SCD, and CIDEC were significantly increased along with PPARγ overexpression in HepG2 cells (Figure 6B), suggesting that PPARγ overexpression enhanced downstream signal transduction. We then treated the infected cells with BSA, FFA, or FFA-GA, as mentioned earlier. Oil Red O and triglyceride quantification indicated that BSA- or FFA-treated cells exhibited more severe lipid deposition when PPARγ was overexpressed (Figure 6C–6E). Moreover, after PPARγ upregulation, the suppressive action of GA on PPARγ expression in HepG2 cells was greatly reversed (Figure 6F), accompanied by higher levels of intracellular triglyceride contents and Oil Red O positive areas (Figure 6C–6E), implying that the protective effects of GA in lipid accumulation were partially abolished in the PPARγ-overexpressing cells. Furthermore, liver-specific overexpression of PPARγ mediated by tail vein injection of the adenovirus carrying pAAV-TBG-Pparg-P2A-EGFP (AAV-PPARG) plasmid vector in HFHC-fed mice significantly diminished the therapeutic effect of GA. GA administration combined with AAV injection significantly increased the body weight, liver weight, serum triglyceride, ALT, and AST levels of HFHC-fed mice compared with the GA treatment group (Figure 7A–F). AAV transfection also abolished the beneficial effects of GA on hepatic steatosis and lipid droplet accumulation (Figure 7G, H). Collectively, these results demonstrate that the ability of GA to attenuate hepatocyte steatosis is dependent on the inhibition of PPARγ signaling pathway.

### 3.6 GA inhibits PPARγ gene expression by upregulating hepatic IRF6

**FIGURE 8:**
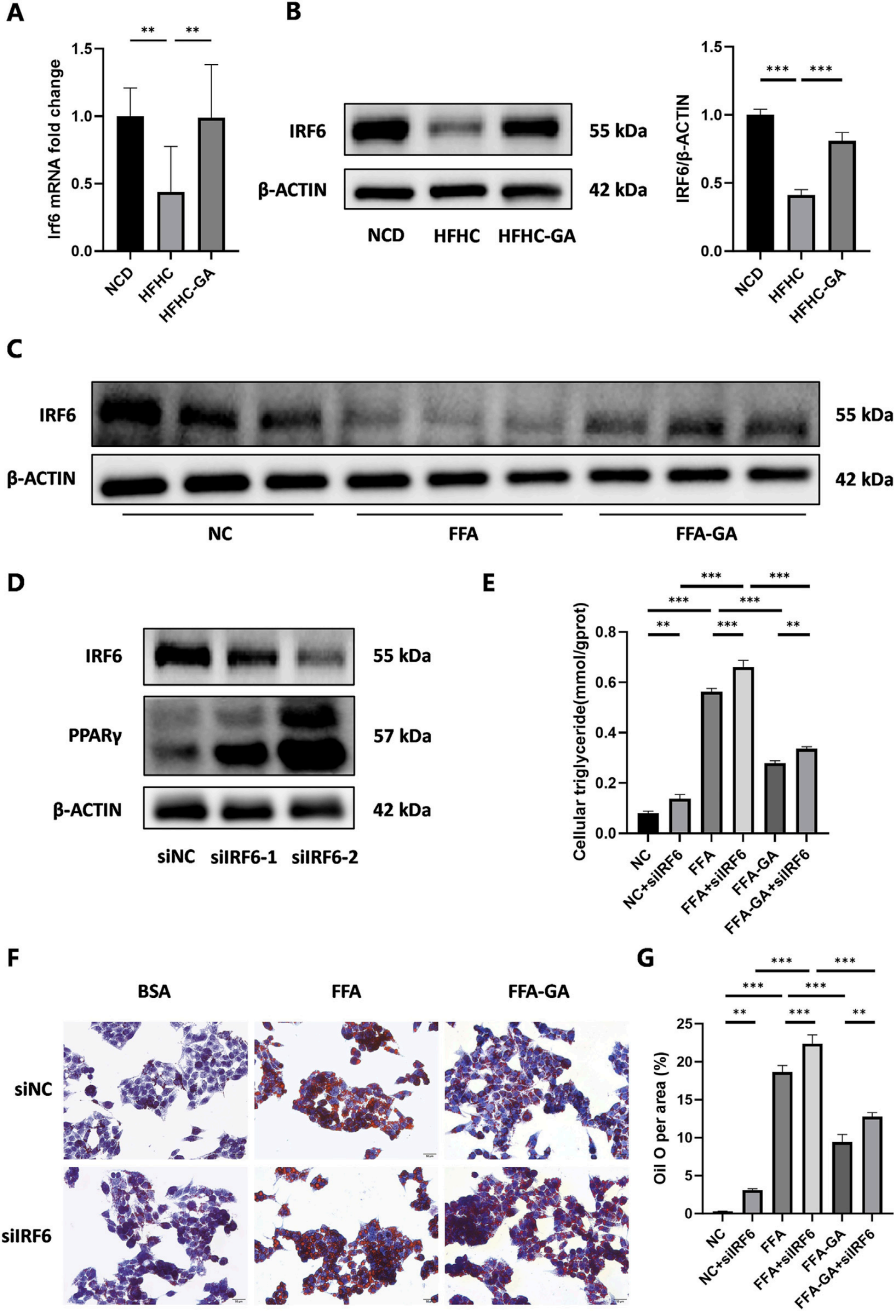
Modulation of hepatic lipid accumulation by GA via the IRF6/PPARγ pathway. **(A,B)** Analysis of expression of IRF6 in the livers of NCD, HFHC, and GA mice by qPCR (n = 3 per group) and Western blot (n = 3 per group). **(C)** Analysis of IRF6 expression by Western blot in HepG2 cells after FFA and FFA-GA treatment for 24 h, respectively (n = 3 per group). **(D)** Analysis of IRF6 and PPARγ expression by Western blot in IRF6-knockdown HepG2 cells (n = 3 per group). **(E)** Triglyceride levels in IRF6-knockdown HepG2 cells treated with FFA and FFA-GA, respectively (n = 3 per group). **(F)** Oil red O staining of IRF6-knockdown HepG2 cells treated with FFA and FFA-GA, respectively (n = 3 per group). **(G)** Quantification of Oil red O positive area per field (n = 3 per group). Scale bar: 100 µm. The data are presented as the mean ± SEM. *p < 0.05, **p < 0.01, ***p < 0.001.

Upstream regulator analysis using IPA revealed that IRF6 was the top enriched upstream regulator (Table 1). Previous studies have demonstrated that hepatic IRF6, a transcription factor, serves as a critical factor in liver steatosis and exerts its role through negative regulation of PPARγ (Tong et al., 2019). To further validate this regulatory relationship, we performed bioinformatic analysis using JASPAR to predict IRF6 binding sites within the PPARγ promoter. Our analysis identified 4 high-confidence IRF6 binding motifs located at positions 2225 to 2233, 1021 to 1029, 2814 to 2822, and 548 to 556 related to the transcription start site (TSS) of PPARγ (Supplementary Table 3), suggesting that IRF6 may directly regulate PPARγ transcription by binding to its promoter. Based on these findings, it is reasonable to infer that GA enhances IRF6 production upon metabolic stimuli exposure, which mediates PPARγ transcriptional regulation. Furthermore, qPCR and Western blot analyses of mouse liver IRF6 in the GA-treated group showed a significant increase compared to the HFHC group (Figure 8A, B). Additionally, IRF6 protein levels in HepG2 cells notably increased after GA treatment (Figure 8C). Thus, we speculated that GA inhibits lipid accumulation by targeting IRF6. To verify this hypothesis, two IRF6-specific siRNAs (siIRF6-1 and siIRF6-2) were constructed to silence endogenous IRF6 expression in HepG2 cells. Western blot analysis revealed that siIRF6-2 displayed a higher knockdown efficiency than siIRF6-1 (Figure 8D), leading to its selection for subsequent experiments. Interestingly, with the elimination of IRF6 in hepatocytes, the protein level of PPARγ observably increased, and the increase exhibited an IRF6 deficiency-dependent manner (Figure 8D), suggesting that silencing IRF6 weakens its inhibitory effect on PPARγ expression. Furthermore, we performed Oil Red O staining and detected cellular triglycerides to assess the effect of IRF6 knockdown on GA-mediated effects. As expected, the GA-induced alleviation of lipid accumulation was significantly blocked after IRF6 knockdown (Figure 8E, F). Collectively, the results demonstrated that GA downregulates the mRNA and protein levels of PPARγ by upregulating IRF6 in hepatocytes.

**TABLE 1:** Upstream regulators of DEPs predicted by IPA.

Upstream Regulator	Molecule Type	Predicted Activation state	Activation z-score	p-value Of overlap
IRF6	Transcription regulator	Activated	3.5	8.04E-09
CITED2	Transcription regulator	Activated	3.12	0.00000021
SIRT1	Transcription regulator	Activated	3.051	1.82E-09
Tcf7	Transcription regulator	Activated	2.63	0.00174
HOXA10	Transcription regulator	Activated	2.449	0.0000371
SREBF2	Transcription regulator	Inhibited	−2.766	6.22E-09
CREB1	Transcription regulator	Inhibited	−2.89	0.00000698
STAT1	Transcription regulator	Inhibited	−2.899	0.000000297
CEBPA	Transcription regulator	Inhibited	−2.929	0.000000107
SREBF1	Transcription regulator	Inhibited	−3.499	1.18E-12

## 4 Discussion

**FIGURE 9:**
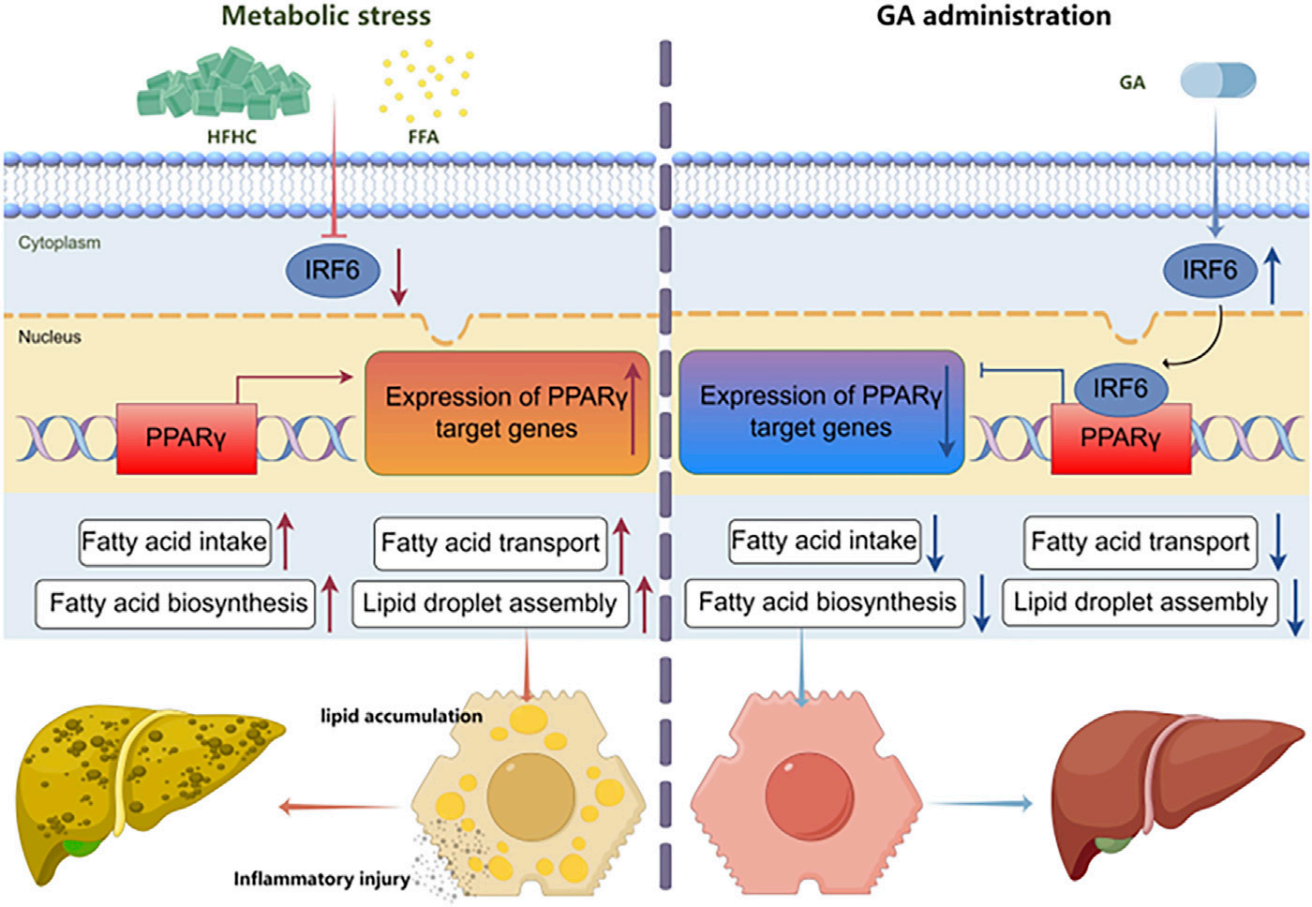
Schematic diagram of the molecular mechanism of GA in treating MASH.

In this study, we explored the protective efficacy of GA in attenuating MASH induced by an HFHC diet in mice. As a regulator of abnormal metabolism, GA reduced the body and liver weights of the HFHC-fed mice. More importantly, GA alleviated IR, hepatic steatosis, inflammation, and liver injury, highlighting its potential therapeutic role in MASH. Mechanistically, integrated RNA sequencing and bioinformatic analysis elucidated that the primary function of GA was to modulate the lipid metabolism of hepatocytes, depending on its transcriptional inhibition of PPARγ signaling, which was subsequently verified through pathway analysis both *in vivo* and *in vitro*. Based on IPA analysis and IRF6 biological activities, IRF6 was identified as a potential target of GA and was subsequently validated both *in vitro* and *in vivo*. Our statistics demonstrated that GA increased hepatic IRF6, thereby enhancing the suppression of PPARγ. The decreased PPARγ level downregulated gene expression of downstream lipogenesis-related factors, leading to reduced lipid accumulation and lipotoxicity in hepatocytes (Figure 9).

Despite significant advances in the understanding of MASH, gaps in knowledge regarding its pathogenesis and progression still limit the development of targeted therapies. Recently, resmetirom became the first drug for the management of MASH with landmark Food and Drug Administration (FDA) conditional approval (Kokkorakis et al., 2024). Nevertheless, there is an unmet clinical need for optimal therapies for MASH. With the increasing recognition of the unique benefits of traditional Chinese medicine (TCM) for metabolic diseases (especially NAFLD and NASH) (2016), researchers are exploring potentially effective and safe therapies using TCMs (Yao et al., 2016). GA, which is abundant in many edible and medicinal plants, has demonstrated robust antioxidant and anti-inflammatory activities in metabolic disorders (Xu et al., 2021). Particularly, the favorable impacts of GA on improving simple fatty liver have been demonstrated in several studies, suggesting its potential to restore lipid homeostasis through reprogramming lipid metabolism, reversing mitochondrial function, and repressing apoptosis and inflammatory response caused by hepatocyte-macrophage crosstalk (Tanaka et al., 2020; Zhang et al., 2023; Lu et al., 2023; Chao et al., 2014). The present study is the first to confirm the protective effects of GA against HFHC-induced MASH in mice. Building on this foundation, we introduced an innovative mechanism in which the management of GA on hepatic lipid metabolism relies on the IRF6/PPARγ signaling pathway. Our study contributes to the elucidation of molecular mechanisms by which GA plays a therapeutic role in MASH.

MASLD is triggered by overexpression of genes associated with lipid metabolism (Perakakis et al., 2020; Zhi et al., 2022). In our RNA-seq results, DEGs were highly enriched in fatty acid metabolic processes and immune system regulation, according to GO enrichment analysis. IPA canonical pathway analysis also revealed that the anti-inflammatory process was activated, whereas the pro-inflammatory process and MASLD-related pathways were inhibited, suggesting that GA exerts therapeutic effects on MASLD by promoting hepatic lipid metabolism and suppressing inflammation (Supplementary Figure 3). Furthermore, KEGG analysis suggested that PPAR signaling pathway might be the target pathway of GA, differing from previous findings that GA acts as an AMPK agonist to modulate lipid metabolism (Tanaka et al., 2020; Zhang et al., 2023; Lu et al., 2023). This discrepancy may be attributed to the weaker contribution of AMPK to GA treatment for MASH compared to that of PPAR. Moreover, the transcription factor IRF6 was predicted as the top enriched upstream regulator of DEPs using IPA, which was further validated using qPCR and Western blot assays. Collectively, our findings suggest that GA mainly acts on fatty acid metabolism, which is closely related to the PPAR signaling pathway, with IRF6 as a potential target.

PPARγ belongs to the family of PPARs, which are ligand-activated nuclear receptors involved in regulating various biological processes, including adipogenesis, lipid metabolism, insulin sensitivity, and inflammation. While PPARγ activation has been proposed as a therapeutic strategy for MASLD due to its ability to promote adipocyte differentiation and improve insulin sensitivity (Qiu et al., 2023), its role in MASLD remains complex and controversial. Excessive PPARγ expression in hepatocytes has been linked to increased lipogenesis and lipid deposition, exacerbating hepatic steatosis and MASH progression (Yu et al., 2003; Zhang et al., 2006). This discrepancy highlights the cell-type-specific roles of PPARγ. Our data support that hepatic overexpression of PPARγ aggravates the worsening of MASH. Conversely, we observed that GA decreased the expression of PPARγ and its target genes, thereby weakening lipid synthesis and accumulation. This aligns with previous reports that targeting hepatocyte PPARγ overexpression may be a promising therapeutic strategy for MASH (Wolf Greenstein et al., 2017; Cordoba-Chacon, 2020). To further validate the mechanism, we used PPARγ-overexpressing HepG2 cells and found that the protective effects of GA were counteracted by PPARγ overexpression. This confirms that GA exerts its anti-lipid deposition effects primarily through downregulation of hepatic PPARγ. Notably, GA did not affect the expression of PPARα or PPARβ, the other two subtypes of PPARs. PPARα is a key regulator in fatty acid oxidation, regulating liver lipid metabolism and coordinating energy balance (Pawlak et al., 2015). Similarly, activation of PPARβ promotes lipid oxidation and energy expenditure while potentially reducing lipid accumulation (Wang et al., 2020). Therefore, GA does not influence hepatic fatty acid oxidation.

Previous studies have highlighted the dampening effect of IRF6 on PPARγ in various cell types, showcasing the therapeutic potential of IRF6/PPARγ regulatory axis for multiple diseases (Huang et al., 2017; Li et al., 2017; Tong et al., 2019). IRF6 acts as a PPARγ co-suppressor and directly binds to and suppresses PPARγ activity in murine cerebrovascular endothelial cells, eventually blocking PPARγ-mediated cerebrovascular endothelial cytoprotection following ischemia (Huang et al., 2017). In marrow-derived macrophages, IRF6 inhibits PPARγ expression through directly binding to interferon-stimulated response elements located upstream of the PPARγ coding region, causing a failure of PPARγ-dependent M2 activation, which makes it a target of dysregulated immunologic homeostasis (Li et al., 2017). Similarly, hepatic IRF6 directly binds to the promoter of the PPARγ gene, thereby transcriptionally suppressing PPARγ under normal conditions. In contrast, during metabolic stress, promoter hypermethylation of IRF6 may silence its gene expression and release PPARγ from repression, causing disturbances to lipid metabolism (Tong et al., 2019). Based on this discovery, our study indicated that GA-mediated IRF6 upregulation strengthens the negative regulatory effect on PPARγ, which is beneficial in reducing liver metabolic abnormalities. Notably, there is currently no research on natural compounds targeting the hepatic IRF6/PPARγ pathway. Our study is the first to identify GA as a suppressor of PPARγ through the upregulation of IRF6 expression. However, the precise mechanism by which GA upregulates IRF6 expression and its binding targets remains unknown and requires further investigation.

## 5 Conclusion

In summary, we validated the efficacy of GA in an HFHC-induced MASH mouse model and explored the potential pathways through which GA modulates hepatic lipid metabolism using RNA-seq analysis in mice. Our results indicated that GA downregulated the PPARγ signaling by increasing hepatic IRF6 levels, which then weakened lipid synthesis and accumulation by downregulating lipogenesis-related factors, ultimately relieving hepatic steatosis and liver damage. Therefore, GA may have the potential to serve as a promising therapeutic option for MASH.

## Data Availability

The data presented in the study are deposited in the Genome Sequence Archive (GSA) repository, accession number CRA024030.

## Glossary

MASLD: Metabolic dysfunction-associated steatotic liver disease
MASH: Metabolic dysfunction-associated steatohepatitis
PPARγ: Peroxisome proliferator-activated receptor gamma
MCD: Methionine/choline deficient diet
IRF6: Interferon regulatory factor 6
GA: Gallic acid
AMPK: AMP-activated protein kinase
RNA-seq: RNA sequencing
HFHC: High-fat high-carbohydrate diet
ITT: Insulin tolerance test
GTT: Glucose tolerance test
HE: Hematoxylin–eosin
HRP: Horseradish peroxidase
DAB: 3,3'-diaminobenzidine
ALT: Alanine aminotransferase
AST: Aspartate aminotransferase
IL-6: Interleukin-6
IL-1β: Interleukin-1β
TNF-α: Tumor necrosis factor-α
IFN-γ: Interferon-γ
IL-17A: Interleukin-17A
ELISA: Enzyme linked immunosorbent assay
CCK-8: Cell counting kit-8
qRT-PCR: Quantitative real-time polymerase chain reaction
SDS-PAGE: Sodium dodecyl sulfate-polyacrylamide gel electrophoresis
LV: Lentivirus
MOI: Multiplicity of infection
siRNA: Small-interfering RNA
SEM: Standard error of mean
MET: Metformin
NCD: Normal chow diet
IR: Insulin resistance
NAS: NAFLD Activity Score
BSA: Bovine serum albumin
PCA: Principal component analysis
DEG: Differentially expressed gene
GO: Gene Ontology
KEGG: Kyoto Encyclopedia of Genes and Genomes
PPI: Protein-protein interaction
CD36: Cluster of Differentiation 36
SCD: Stearoyl Coenzyme A Desaturase
FABP2: Fatty Acid Binding Protein 2
FASN: Fatty Acid Synthase
FADS2: Fatty Acid Desaturase 2
CIDEC: Cell Death Inducing DFFA Like Effector C
ME1: Malic Enzyme 1
PLTP: Phospholipid Transfer Protein
IPA: Ingenuity Pathway Analysis
FDA: Food and Drug Administration
TCM: Traditional Chinese medicine
