# Supplementary material for: Gallic acid mitigates high-fat and high-carbohydrate diet-induced steatohepatitis by modulating the IRF6/PPARγ signaling pathway
Source: Front Pharmacol. 2025 Apr 1;16:1563561. doi: 10.3389/fphar.2025.1563561 (PMC11996808; doi:10.3389/fphar.2025.1563561)
Supplement: Supplementary file 2 [file DataSheet1.docx]

**Supplementary materials**

**Gallic acid mitigates** **high-fat and high-carbohydrate diet-induced steatohepatitis by modulating the IRF6/PPARγ signaling pathway**

Jiahao Qiu ^a, b, 1^, Lihong Fu ^b, 1^, Yan Xue ^a, b, 1^, Yanlu Ma ^b^, Yilan Yang ^b^, Fengjie Qiao ^b^, Wanchun Zhu ^b^, Yating Gao ^b^, Miao Fang ^b^, Yufei Liu ^d^, Zhujun Gao ^d^, Yunfeng Guan ^a, b^, Yueqiu Gao ^a, b, c*^, Xin Zhang ^b*^, Zhi Shang ^a, b, c*^

^a^ Institute of Infectious Disease, Shuguang Hospital Affiliated to Shanghai Universi ty of Traditional Chinese Medicine, Shanghai, China

^b^ Laboratory of Cellular Immunity, Shuguang Hospital Affiliated to Shanghai University of Traditional Chinese Medicine, Shanghai, China

^c^ Department of Liver Diseases, Shuguang Hospital Affiliated to Shanghai University of Traditional Chinese Medicine, Shanghai, China

^d^ Shanghai University of Traditional Chinese Medicine, Shanghai, China

Authorship note: ^1^ These authors contributed equally to this study.

*Corresponding authors: Zhi Shang, PhD, Institute of Infectious Disease, Shuguang Hospital Affiliated to Shanghai University of Traditional Chinese Medicine, 528 Zhangheng Road, Shanghai, China, 201203. Telephone: +86 21 20256217. E-mail: shangzhi90@gmail.com; or Yueqiu Gao, MD, gaoyueqiu@hotmail.com; or Xin Zhang, MD, [zhangxin68619@163.com](mailto:zhangxin68619@163.com).

**Supplementary Table 1. Primer pairs for qRT-PCR detection.**

| Gene | Sequence 5'→3' |
| --- | --- |
| mouse *Fasn* | F: GGAGGTGGTGATAGCCGGTAT |
|  | R: TGGGTAATCCATAGAGCCCAG |
| mouse *Pparγ* | F: ATTCTGGCCCACCAACTTCGG |
|  | R: TGGAAGCCTGATGCTTTATCCCCA |
| mouse *Scd1* | F: TCTTCCTTATCATTGCCAACACCA |
|  | R: GCGTTGAGCACCAGAGTGTATCG |
| mouse *Fabp2* | F: ATGGCGTTTGACAGCACTTG |
|  | R: TCAGTTCCGTCTGCTAGATTGTA |
| mouse *Fads2* | F: TCATCGGACACTATTCGGGAG |
|  | R: GGGCCAGCTCACCAATCAG |
| mouse *Cidec* | F: TGTCGTGTTAGCACCGCAG |
|  | R: TTGCGCTGTTCTGATGGGG |
| mouse *Cd36* | F: GACTGGGACCATTGGTGATGA  R: AAGGCCATCTCTACCATGCC |
| mouse *Irf6* | F: CTCCACCCTCGAAGAGTCC |
|  | R: AGCTGGGTCAGGATCGTCTAC |
| mouse *Pparα* | F: TATTCGGCTGAAGCTGGTGTAC |
|  | R: CTGGCATTTGTTCCGGTTCT |
| mouse *Pparβ* | F: TCCATCGTCAACAAAGACGGG |
|  | R: ACTTGGGCTCAATGATGTCAC |
| mouse *Me1* | F: CTGCTGACACGGAACCCTC |
|  | R: GATCTCCTGACTGTTGAAGGAAG |
| mouse *Pltp* | F: CGCAAAGGGCCACTTTTACTA |
|  | R: GCCCCCATCATATAAGAACCAG |
| mouse *Actb* | F: GGCTGTATTCCCCTCCATCG  R: CCAGTTGGTAACAATGCCATGT |

**Supplementary Table 2. Primary antibodies for western blot assays.**

| Antibody name | Cat No. | Manufacturer |
| --- | --- | --- |
| PPAR Gamma Monoclonal antibody | 66936-1-Ig | Proteintech |
| PPARA Monoclonal antibody | 66826-1-Ig | Proteintech |
| PPARD Monoclonal antibody | 60193-1-Ig | Proteintech |
| FASN Polyclonal antibody  SCD Polyclonal antibody  FABP2 Polyclonal antibody | 10624-2-AP  28678-1-AP  21252-1-AP | Proteintech  Proteintech  Proteintech |
| CD36 Polyclonal antibody  CIDEC Polyclonal antibody  IRF6 Polyclonal antibody | 18836-1-AP  12287-1-AP  12928-1-AP | Proteintech  Proteintech  Proteintech |
| Beta Actin Monoclonal antibody | 66009-1-Ig | Proteintech |

**Supplementary Table 3. High-confidence IRF6 binding motifs.**

| Score | Relative score | Start | End | Strand | Predicted sequence |
| --- | --- | --- | --- | --- | --- |
| 4.7049403 | 0.80913574 | 548 | 556 | + | ACTAAAACT |
| 5.206612 | 0.8172473 | 2814 | 2822 | - | GCCGAGACC |
| 5.443147 | 0.8210718 | 1021 | 1029 | - | TCTGAAACT |
| 5.7029953 | 0.8252733 | 2225 | 2233 | + | CCCTAAACT |

**
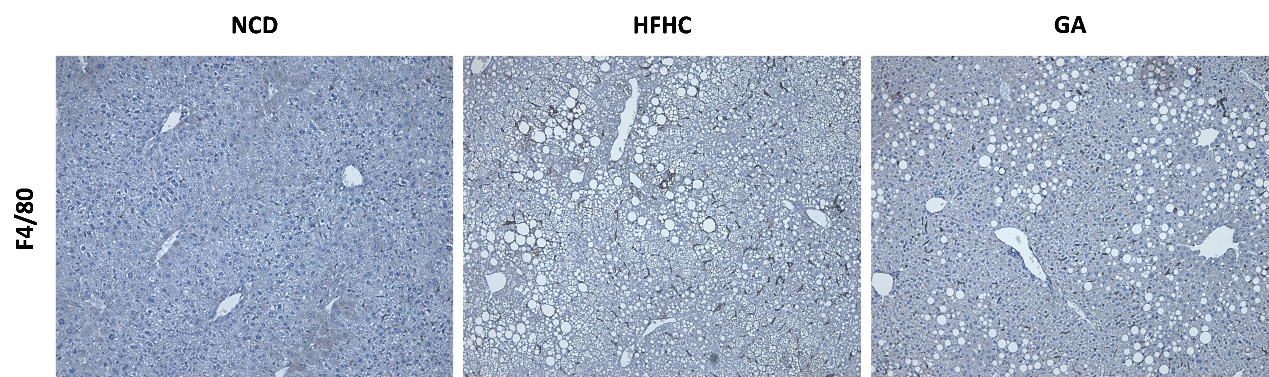
**

**Supplementary Fig 1.** Immunohistochemical staining of F4/80 of liver sections (n = 3).


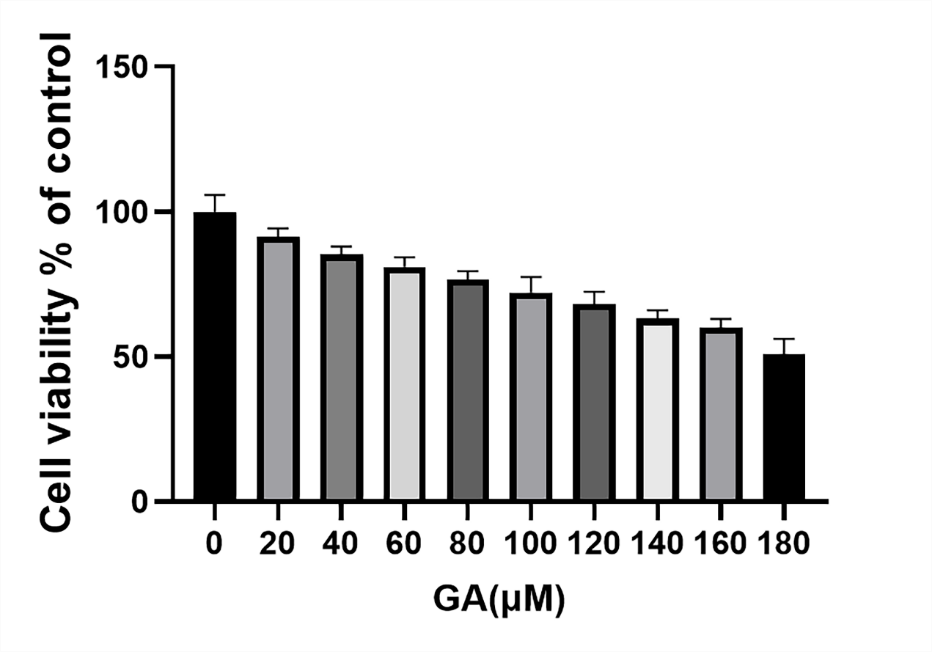


**Supplementary Fig 2.** Cell viability of HepG2 cells treated with different concentration of GA (n = 3).


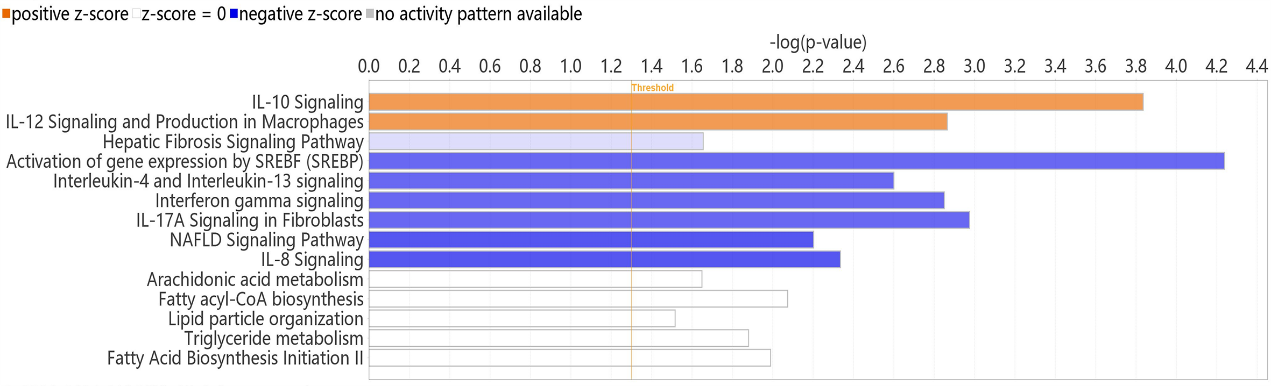


**Supplementary Fig 3.** IPA canonical pathway analysis of DEGs.
